# The role of major depression in neurocognitive functioning in patients with posttraumatic stress disorder

**DOI:** 10.3402/ejpt.v4i0.19979

**Published:** 2013-05-02

**Authors:** Mirjam J. Nijdam, Berthold P. R. Gersons, Miranda Olff

**Affiliations:** 1 Department of Psychiatry, Academic Medical Center at the University of Amsterdam, Amsterdam, The Netherlands; 2 Arq Psychotrauma Expert Group, Diemen, The Netherlands

**Keywords:** neuropsychology, cognitive functioning, PTSD, major depressive disorder, comorbidity

## Abstract

**Background:**

Posttraumatic stress disorder (PTSD) and major depressive disorder (MDD) frequently co-occur after traumatic experiences and share neurocognitive disturbances in verbal memory and executive functioning. However, few attempts have been made to systematically assess the role of a comorbid MDD diagnosis in neuropsychological studies in PTSD.

**Objective:**

The purpose of the current study is to investigate neurocognitive deficits in PTSD patients with and without MDD. We hypothesized that PTSD patients with comorbid MDD (PTSD+MDD) would have significantly lower performance on measures of verbal memory and executive functioning than PTSD patients without MDD (PTSD–MDD).

**Method:**

Participants included in this study were 140 treatment-seeking outpatients who had a diagnosis of PTSD after various single traumatic events and participated in a randomized controlled trial comparing different treatment types. Baseline neuropsychological data were compared between patients with PTSD+MDD (*n*=84) and patients with PTSD–MDD (*n*=56).

**Results:**

The PTSD+MDD patients had more severe verbal memory deficits in learning and retrieving words than patients with PTSD alone. There were no differences between the groups in recall of a coherent paragraph, recognition, shifting of attention, and cognitive interference.

**Conclusions:**

The results of this study suggest that a more impaired neurocognitive profile may be associated with the presence of comorbid MDD, with medium-sized group differences for verbal memory but not for executive functioning. From a clinical standpoint, being aware that certain verbal memory functions are more restricted in patients with comorbid PTSD and MDD may be relevant for treatment outcome of trauma-focused psychotherapy.

Posttraumatic stress disorder (PTSD) and major depressive disorder (MDD) are common outcomes after experiencing a traumatic event (Kessler, Sonnega, Bromet, Hughes, & Nelson, 1995), with considerable overlap in their symptomatology. Sleep disturbances, diminished interest in activities, and impaired concentration are shared diagnostic criteria of these disorders in DSM-IV. Therefore, it is not surprising that the comorbidity of these disorders is high; approximately 50% of PTSD patients also meet criteria for MDD (Kessler et al., 1995; Shalev et al., 1998). Some investigators see symptoms diagnosed as comorbid depression as reflecting the construct of PTSD (O'Donnell, Creamer, & Pattison, 2004), whereas others have found support for separating core PTSD symptoms from dysphoria symptoms (Rademaker et al., 2012).

Neuropsychological studies have consistently confirmed disturbances in concentration or sustained attention in different trauma populations with PTSD (Horner and Hamner, 2003), as well as consistent deficits in verbal memory (Brewin, Kleiner, Vasterling, & Field, 2007) and executive functioning (Polak, Witteveen, Reitsma, & Olff, 2012). Major depression in itself is also associated with several neurocognitive deficits, which are most pronounced in mental flexibility, control, and effortful processing (Veiel, 1997). These neuropsychological disturbances can be measured in a way that closely approximates the difficulties that patients face in their daily lives, such as remembering a grocery list, summarizing a newsflash they just heard on the radio, inhibiting irrelevant information while performing a certain task, and performing multiple tasks at a time.

In PTSD populations, few attempts have been made to systematically assess the role of a comorbid MDD diagnosis in neuropsychological studies. So far, in most studies the severity of depressive symptoms has been examined instead of the diagnosis. Sample sizes in these studies were mostly small and therefore have an increased risk of false–negative findings. Sachinvala et al. (2000) investigated the role of depressive symptoms in neuropsychological performance of Vietnam veterans with chronic PTSD and found that depressive symptoms negatively correlated with memory performance, but not with attention. A recent study in asylum seekers confirmed that verbal memory deficits specifically, and not other neuropsychological deficits, are related to depressive symptoms in PTSD (Johnsen, Kanagaratnam, & Asbjørnsen, 2008). However, a meta-analysis of studies on executive function in PTSD found that divided attention and working memory were significantly related to the severity of comorbid depressive symptoms in PTSD populations, whereas selective attention and interference were not (Polak et al., 2012).

The purpose of the current study is to investigate neurocognitive deficits in PTSD patients with and without MDD. Knowing more about these deficits can help us understand whether PTSD and comorbid MDD are part of the same construct, as well as have potential clinical relevance regarding treatment of PTSD (Wild & Gur, 2008). Neurocognitive tests in this study were selected based on their close approximation of the everyday difficulties that patients with PTSD and MDD face. Based on the previously mentioned studies, we hypothesized that those PTSD patients with comorbid MDD would have a significantly lower performance on tests of verbal memory and executive functioning than PTSD patients without MDD.

## Method

### Participants

Participants included in this study were 140 outpatients who had a diagnosis of PTSD after various single traumatic events who sought treatment at the Academic Medical Center at the University of Amsterdam. They agreed to participate in a randomized clinical trial comparing the effects of two forms of trauma-focused psychotherapy. Further details about the randomized controlled trial are described elsewhere (Nijdam, Gersons, Reitsma, De Jongh & Olff, 2012). The PTSD+MDD group consisted of 84 patients who met criteria for a major depressive disorder at baseline, and the PTSD–MDD group consisted of 56 patients who did not meet the criteria for a major depressive disorder at baseline. In this article, we report on data of the first assessment, which took place before patients were randomized and received treatment.

Study inclusion criteria were: 1) a PTSD diagnosis according to DSM-IV; 2) a single traumatic event that led to the development of PTSD and had stopped at the time of inclusion; 3) age between 18 and 65 years; 4) mastery of the Dutch language. Exclusion criteria were: 1) acute suicidality; 2) current severe MDD or current severe alcohol or substance dependence according to DSM-IV; 3) a lifetime psychotic disorder according to DSM-IV; and 4) a severe personality disorder according to the SCID-II screener (First, Gibbon, Spitzer, Williams, & Benjamin, 1997) and DSM-IV (classified as severe if more than the required number of symptoms were present and the disorder persistently influenced the person in multiple areas of functioning over a prolonged period in life). Patients with a history of earlier traumatic experiences were allowed to participate in the trial. For patients on parallel pharmacological treatment, a stable regimen for at least four weeks was required before entering the study.

### Measures

#### Clinical measures

PTSD diagnoses were established by means of the Structured Interview for PTSD (Davidson, Malik, & Travers, 1997), which operationalizes the DSM-IV criteria for PTSD and measures their frequency and severity. MDD diagnoses and other comorbid psychiatric diagnoses were assessed with the Structured Clinical Interview for DSM-IV Disorders (SCID-I/P; Spitzer, Gibbon, Janet M, & Janet W, 1996). A Dutch version of the Impact of Event Scale–Revised (IES-R) was used as a self-report of PTSD symptom severity (Weiss & Marmar, 1997). Unlike the original revised version in which categories from 0–4 are used, this Dutch IES-R rates the frequency of each item in the preceding week as 0 (= *not at all*), 1 (= *rarely*), 3 (= *sometimes*), and 5 (= *often*) and the total PTSD score (range 0–110) consists of the sum of the scores. The depression scale of the Hospital Anxiety and Depression Scale (HADS) was used to measure the severity of the depressive symptoms by self-report (Zigmond & Snaith, 1983). All of these measures have been widely used in trauma research and have been shown to have good psychometric properties (Creamer, Bell, & Failla, 2003; Davidson et al., 1997; Spinhoven et al., 1997; Zanarini & Frankenburg, 2001).

#### Neuropsychological measures

The California Verbal Learning Test (CVLT; Delis, Kramer, Kaplan, & Ober, 1987) is a multi-trial serial learning test that measures encoding, short-term retrieval, long-term retrieval, and recognition of verbal information. A grocery list of 16 items is presented five times (List A), and patients are instructed to recall as many items as possible after each presentation. The sum of correct responses on these first five trials is a measure of encoding performance (range of correct responses 0–80). After a distracting list (List B), patients are asked to recall List A at once (short-term retrieval; range 0–16) and after an interval of 20 min (long-term retrieval; range 0–16). Cued retrieval is measured by giving semantic cues to enhance recall, measured both immediately (short-term cued retrieval) and after a 20 min interval (long-term cued retrieval). Recognition memory is measured on a 44-item list including items of List A and B, and unfamiliar words; patients are asked to identify whether the word was part of List A or not (range of correct responses 0–44). Psychometric properties of the CVLT are sufficient (Paolo, Tröster, & Ryan, 1997).

The Paragraph Recall Subtest of the Rivermead Behavioural Memory Test (RBMT; Wilson, Cockburn, & Baddeley, 1985) is a test of short-term and long-term verbal memory. It is a test of everyday memory consisting of two newspaper excerpts read out loud to the patient. The patient is asked to recall the excerpt directly after hearing it (short-term retrieval) and after a 15 min interval (long-term retrieval). The sum of correctly recalled items determines the test score (range 0–42). The RBMT has shown to be a valid and reliable indicator of memory impairment in various populations (Wilson, Cockburn, Baddeley, & Hiorns, 1989).

The Trail Making Test (TMT) is a test to measure shift of attention, planning and cognitive flexibility (Reitan, 1955). Patients are asked to track a number sequence on a paper sheet (Part A) and a sequence of alternating numbers and letters (Part B) as fast as possible. The required time in seconds is measured and constitutes the score on the test. The time needed to complete part A and part B are both measures of mental speed, with part B focusing more on alternated attention. Reliability and validity of the TMT is high (Lezak, 1995).

The Stroop Color Word Test is thought to measure selective attention and cognitive flexibility (Homack & Riccio, 2004). This well-known test consists of three trials. With the first card, patients are asked to read out loud colour names printed in black ink. With the second card, they are asked to name blocks of the same colours. On the third card, the colour names are printed in incongruent ink, and patients are asked to name the colour of the ink. The interference score is calculated by the time in seconds used to complete the third card, minus the time in seconds on the second card. The reliability of the Stroop Color Word Test is sufficient (Strauss, Allen, Jorgensen, & Cramer, 2005).

### Procedure

At the start of the assessments, the procedure of the study was fully explained, after which patients were asked to participate and give their written informed consent. Psychologists or master's level psychology students under the supervision of an experienced psychologist carried out assessments. Patient confidentiality was maintained. The Institutional Medical Ethics Committee of the Academic Medical Center approved this study.

### Statistical analyses

Analyses were conducted using SPSS version 19.0 (IBM SPSS, USA). Chi-square tests and independent *t*-tests were used to compare baseline demographic and clinical characteristics between the two groups. Square root and square transformations were performed if distributions significantly departed from the normal distribution curve. Square transformations were performed for the CVLT variables and square root transformations for TMT and Stroop variables. Two outliers were excluded from the analysis of CVLT variables and one outlier from the analysis of TMT and Stroop variables. Mean scores on all outcome variables per neuropsychological test were analysed in a multivariate general linear model as a function of the presence of MDD (two levels). If this overall test was significant, we examined group differences on the separate test variables within the univariate general linear model. Two-tailed tests were applied throughout and level of significance was set at *α*=0.05 for the multivariate tests. Level of significance for the univariate analyses was set at *α*=0.01 to correct for multiple testing. Partial-eta squared was calculated as an effect size for significant differences.

## Results

### Demographic and clinical characteristics

Demographic and clinical characteristics of the two groups are displayed in Table 1. No significant differences emerged on demographic variables or clinical features between the PTSD+MDD and PTSD–MDD groups, except that the PTSD+MDD group had significantly more severe PTSD symptoms on the IES-R and significantly more severe depressive symptoms on the HADS than the PTSD–MDD group (Table 1).

**Table 1:** Demographic and clinical characteristics of the PTSD+MDD and PTSD–MDD group

	PTSD+MDD (*n*=84)		PTSD–MDD (*n*=56)		Analysis		
			
Characteristic	*n*	%	*n*	%	*χ* ^*2*^	df	*p*
Female	50	59.5	29	51.8	0.82	1	0.37
Education					1.53	2	0.47
Low	19	22.6	12	21.4			
Middle	43	51.2	24	42.9			
High	22	26.2	20	35.7			
Dutch	40	47.6	33	58.9	1.72	1	.19
Clinical features							
Type of trauma					6.12^a^		.29
Assault	39	46.4	35	62.5			
Sexual assault	11	13.1	5	8.9			
Accident	20	23.8	6	10.7			
Disaster	5	6.0	5	8.9			
War-related	4	4.8	3	5.3			
Other	5	6.0	2	3.5			
Earlier traumatic experiences	48	57.1	28	50.0	0.52	1	0.47
Anxiety disorder other than PTSD (SCID-I)	13	15.5	9	16.1	0.01	1	0.92
Lifetime alcohol-related disorder (SCID-I)	3	3.6	1	1.8			0.65^a^
Lifetime substance-related disorder (SCID-I)	2	2.4	3	5.4			0.39^a^
On psychoactive medication	39	46.4	20	35.7	1.58	1	0.22
On SRRI	18	21.4	7	12.5	1.83	1	0.18
	Mean	S.D.	Mean	S.D.	*t*	df	*p*
Age	37.32	11.26	38.52	11.58	0.61	138	0.54
Time since trauma (months)	25.17	44.99	37.89	69.87	1.31	138	0.19
PTSD total score (IES-R)	81.98	16.61	68.07	19.75	4.48	137	*<0.001*
Depression severity score (HADS)	13.20	3.85	8.98	3.10	6.85	137	*<0.001*

SCID-I, Structured Clinical Interview for DSM-IV Disorders; PTSD, posttraumatic stress disorder; MDD, major depressive disorder; SSRI, selective serotonin reuptake inhibitor; IES-R, Impact of Event Scale–Revised; HADS, Hospital Anxiety and Depression Scale.

a Fisher's exact test.

### Neurocognitive performance

Mean scores and standard deviations of the two groups on the neurocognitive tests are displayed in Table 2. The multivariate *F*-test revealed a significant difference between the groups for the CVLT, *F*(6,130)=2.89, *p*=0.011. Univariate analyses revealed that the PTSD+MDD group scored significantly lower on CVLT sum of trials 1–5, *F*(1,135)=8.63, *p*=0.004, *η* ^*2*^=0.058; CVLT short-term cued recall, *F*(1,135)=8.15, *p*=0.005, *η* ^*2*^=0.057; CVLT long-term free recall, *F*(1,135)=10.98, *p*=0.001, *η* ^*2*^=0.069; and CVLT long-term cued recall, *F*(1,135)=7.77, *p*=0.006, *η* ^*2*^=0.053, than the PTSD–MDD group. Multivariate *F*-tests did not reveal significant differences between the groups for the RBMT, *F*(2,136)=2.90, *p*=0.059; TMT, *F*(2,135)=1.10, *p*=0.337; or Stroop, *F*(3,134)=1.88, *p*=0.137.

**Table 2:** Means and standard deviations on neurocognitive measures for PTSD+MDD and PTSD–MDD groups

	PTSD+MDD (*n*=83)^a^		PTSD–MDD (*n*=56)	
		
Measure	Mean	SD	Mean	SD
CVLT^b^				
Sum of trials 1–5	47.3	10.8	52.5	9.6
Short-term free recall	10.5	3.0	11.5	3.0
Short-term cued recall	11.3	2.7	12.7	2.5
Long-term free recall	10.7	3.1	12.3	3.1
Long-term cued recall	11.6	2.8	12.8	2.8
Long-term recognition	41.3	3.0	41.5	3.3
RBMT				
Immediate recall	15.2	6.0	17.3	5.9
Delayed recall	11.4	5.6	13.8	6.0
TMT				
Trail A	37.5	23.1	34.0	13.2
Trail B	88.8	40.4	79.0	37.9
Stroop				
Card 2	69.6	24.8	62.6	17.3
Card 3	115.8	66.2	95.1	28.7
Interference	46.3	45.6	33.2	18.4

PTSD, posttraumatic stress disorder; MDD, major depressive disorder; CVLT, California Verbal Learning Test; RBMT, Rivermead Behavioural Memory Test; TMT, Trail Making Test.

a One participant did not complete the neuropsychological assessment at baseline.

b A significant overall group difference was found for the CVLT, *p*=0.011, as well as group differences for CVLT sum of trials 1–5, *p*=0.004, CVLT short-term cued recall, *p*=0.005, CVLT long-term free recall, *p*=0.001, and CVLT long-term cued recall, *p*=0.006.

## Discussion

The main finding of the current study is that treatment-seeking patients with PTSD and comorbid MDD have more pronounced verbal memory deficits than patients with PTSD alone, with medium-sized differences between the groups. These verbal memory deficits in the group with PTSD and MDD seem to be restricted to the encoding, short-term retrieval and long-term retrieval of separate words, since there were no significant differences in retrieval of a coherent paragraph or in recognition between the two groups. Various aspects of executive functioning in the present study were similar for both groups as well, as expressed by a lack of differences on measures of mental speed, shift of attention, selective attention, and cognitive interference. In sum, encoding and retrieval of separate words was a bigger challenge for PTSD patients who had comorbid MDD diagnoses, than for those who had PTSD without MDD. Results of this study thus suggest a somewhat more impaired neurocognitive profile for PTSD patients with comorbid MDD.

Our results confirm and extend the studies of Sachinvala et al. (2000) and Johnsen et al. (2008), which found verbal memory deficits, but not other neurocognitive performance, to be related to the severity of depressive symptoms in various PTSD populations. The results of the current study partly agree with the meta-analytic findings of Polak et al. (2012), who found that comorbid depressive symptoms were not related to selective attention and interference in PTSD populations. However, we found no evidence of an association between comorbid depressive symptoms and shift of attention as found by this meta-analysis. The results of our study are also in contrast with the neuropsychological disturbances found in MDD patients, which are most clear in the domain of mental flexibility and control (Veiel, 1997). Possibly, a combination of PTSD and comorbid MDD leads to a different neurocognitive profile than a diagnosis of MDD alone.

We note that the group with PTSD and comorbid MDD in our study also had more severe PTSD symptoms than the group without comorbid MDD. Therefore, it is possible that the differences we found between the groups are partly attributable to greater PTSD symptom severity. Moreover, it could be the case that PTSD symptom severity contributes to the other neuropsychological processes (e.g., retrieval of a coherent paragraph, shifting of attention, cognitive interference) for which we were not able to confirm statistically significant group differences. However, more severe PTSD symptoms and a comorbid MDD diagnosis are such intertwined constructs that it is very difficult, and may not even be clinically meaningful, to statistically separate the influence of these two variables (Miller & Chapman, 2001). Even though one may discuss whether this is clinically meaningful, inserting PTSD symptom severity as a covariate in our analyses still yielded significant differences on verbal memory between PTSD patients with and without MDD.

Limitations of this study include that we did not administer tests for all types of attention and that we had to exclude patients with severe MDD, as this was an exclusion criterion for the treatment trial. It would be interesting to examine especially sustained attention in samples with PTSD+MDD versus PTSD alone, as depressive symptoms were also found to play a role in sustained attention in PTSD (Meewisse et al., 2005). As our sample consisted of PTSD patients seeking help for single traumatic events, results are not necessarily representative for other PTSD populations. Though more than half of the patients in the current study had experienced multiple traumatic events or early life trauma besides the single traumatic event for which they sought help, results may not extrapolate to survivors with PTSD and MDD resulting from more chronic traumatic experiences. Replication and extension of the neuropsychological findings of the current study is therefore much encouraged, preferably also in trauma survivors who developed MDD on its own after traumatic experiences. It would also be interesting to explore the link between PTSD, MDD, and neuropsychology by investigating associations between neurocognitive impairment and specific PTSD symptoms that do or do not overlap with comorbid MDD (Brewin et al., 2007).

In conclusion, patients with PTSD and comorbid MDD seem to have more difficulty in learning separate units of verbal information and retrieving this information in the short and long term than PTSD patients without comorbid MDD, even when the severity of the MDD diagnosis is only mild to moderate. PTSD symptom severity may contribute to these difficulties. From a clinical standpoint, it is good to be aware that these aspects of verbal memory are more restricted in patients with comorbid PTSD and MDD, as verbal memory performance can influence the treatment outcome of trauma-focused psychotherapy (Wild & Gur, 2008; Nijdam, De Vries, Gersons & Olff, submitted). The clinical relevance of the differences we found between PTSD patients with and without MDD, with medium effect sizes, requires further study. These neuropsychological deficits may help us to determine subgroups of PTSD patients with different treatment prognoses and in the future possibly more targeted interventions.
